# Supplementary material for: Ligand-specific conformational change drives interdomain allostery in Pin1
Source: Nat Commun. 2022 Aug 4;13:4546. doi: 10.1038/s41467-022-32340-x (PMC9352728; doi:10.1038/s41467-022-32340-x)
Supplement: Supplementary file 1 — Supplementary Information [file 41467_2022_32340_MOESM1_ESM.pdf]

Ligand-specific conformational change drives interdomain allostery in Pin1

Supplementary Information

SI: Results and Discussion

After ligand addition, the distance between the intradomain (PPIase) positions 90-131 could be assessed by the 4pDEER experiment, whereas for most of the longer interdomain distances between the WW and PPIase domain, namely mutants 15-98, 15-90 and 15-131, the 5pDEER experiment was required (Supplementary Figure 3). For 15-98 and 15-90, the sum of two Gaussian components (2Gauss) models the time-domain DEER data equally well as the unparametrized distance distribution obtained by Tikhonov regularization (Supplementary Table 2 and 3). Instead, for 90-131 and 15-131 only the unparametrized distance distributions, obtained by either Tikhonov regularization or a neural network approach, provide an adequate description of the DEER data (Supplementary Table 2 and 4). Among these two approaches, Tikhonov regularization provides better fits of the time-domain data for positions 90-131 (Supplementary Figure 3), albeit at the cost of modelling the data by a large number of unrealistically narrow peaks. The problem could be traced back to excitation band overlap between observer and pump pulses, which, for such a narrow distance distribution, causes contributions from additional coherence transfer pathways that cannot be fully removed by cutting off data[1]. Neural network analysis by DEERNet[2] is less susceptible to distortions due to these additional signals, as it inherits information on realistic numbers of peaks from the data sets used in training the neural networks. Therefore, we opted for showing the DEERNet distributions in the main text. As seen in Supplementary Figure 3, the main features of the distance distributions do not differ between Tikhonov regularization and neural network analysis and we used only these for drawing conclusions. Note that when combining the latter mutant with the ligand FFpSPR (Supplementary Figure 3a), the dipolar oscillations of 5pDEER traces coincide with the refocusing time of the 4pDEER pathway (“artefact”).

Supplementary Figures

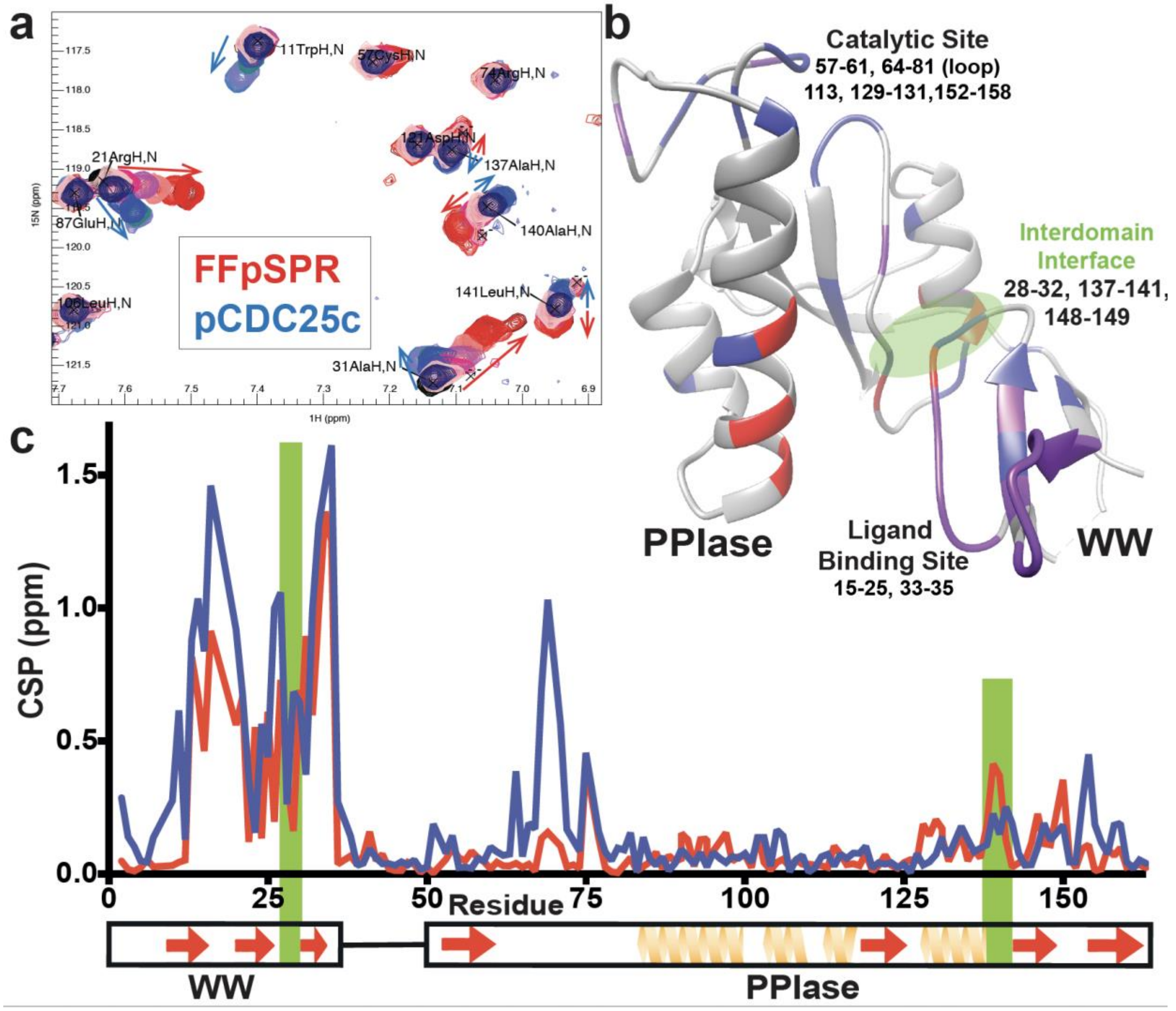

Supplementary Figure 1. **Interdomain allostery probed by chemical shift perturbations (CSPs) of Pin1 binding to FFpSPR and pCDC25c. a** Example of ligand-specific CSPs mainly in the interdomain (ID) interface. CSPs plotted **b** on the structure of PDB entry 1pin[3] and **c** per residue. CSPs were calculated using equation $\Delta\boldsymbol{\delta} = \sqrt{\Delta\boldsymbol{\delta}_{\boldsymbol{H}}{}^{2} + \mathbf{0.15}(\Delta\boldsymbol{\delta}_{\boldsymbol{N}}{}^{2})}$. Major CSPs plotted onto the structure used cutoffs of 0.5 and 0.15 ppm for the WW and PPIase domains, respectively. Major CSPs in both FFpSPR and pCDC25c titrations are colored purple in b. Important residues are annotated in b, with the ID interface shaded in green in b and c.

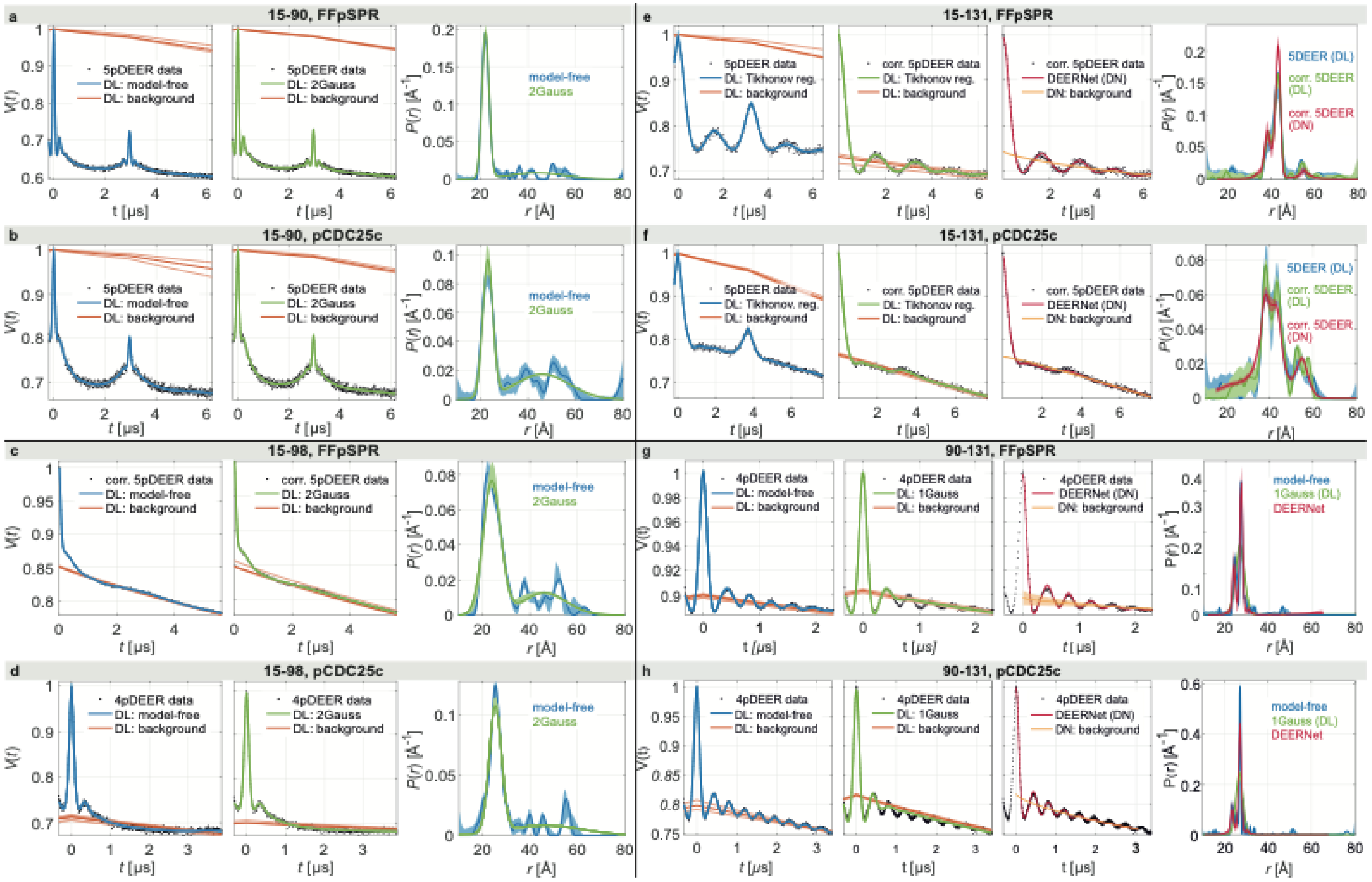

a
15-90, FFpSPR
5pDEER data
DL: model-free
DL: background
DL: 2Gauss
model-free
2Gauss
b
15-90, pCDC25c
c
15-98, FFpSPR
corr. 5pDEER data
d
15-98, pCDC25c
4pDEER data
e
15-131, FFpSPR
DL: Tikhonov reg.
DEERNet (DN)
DN: background
5DEER (DL)
corr. 5DEER (DL)
corr. 5DEER (DN)
f
15-131, pCDC25c
g
90-131, FFpSPR
DL: 1Gauss
1Gauss (DL)
DEERNet
h
90-131, pCDC25c
V(t)
P(r) [Å^-1]
t [μs]
r [Å]

Supplementary Figure 2. **Analysis of the DEER data.** DEER data $V(t)$ of ligand-bound mutants was analyzed with DeerLab (DL) and/or DEERNet (DN, Spinach SVN Rev 5662) using either the 5pDEER or 4pDEER model and a stretched exponential background (orange). For 15-90 and 15-98 mutants (**a**-**d**), either a model-free (left, blue) or sum of two Gaussian distance distribution (2Gauss) $P(r)$ (middle, green) was used. For ligand-bound 15-131 mutant (**e**, **f**), the 5pDEER data $V(t)$ was analyzed using the 5pDEER model (ex_5pdeer; left, blue), the corrected 5pDEER using the 4pDEER model (ex_4pdeer; second from left, green) combined with a stretched exponential background (orange) and a model-free distance distribution, and neural network analysis by DEERNet (third from left, red). For ligand-bound 90-131 mutant, (**g**, **h**) the 4pDEER data $V(t)$ was analyzed using the ex_4pdeer model, a stretched exponential background (orange) and either a model-free (left, blue) or a Gaussian distance distribution (1Gauss) $P(r)$ (second from left, green), and neural network analysis by DEERNet (third from left, red). The corresponding $P(r)$ are compared on the right for a) 15-90 FFpSPR, b) 15-90 pCDC25c, c) 15-98 FFpSPR, d) 15-98 pCDC25c, e) 15-131 FFpSPR, f) 15-131 pCDC25c, g) 90-131 FFpSPR and h) 90-131, pCDC25c, presented as mean values with shaded areas representing the 95% confidence interval. See Supplementary Table 2-4 for the parameters of the model-free and Gaussian $P(r)$ fits.

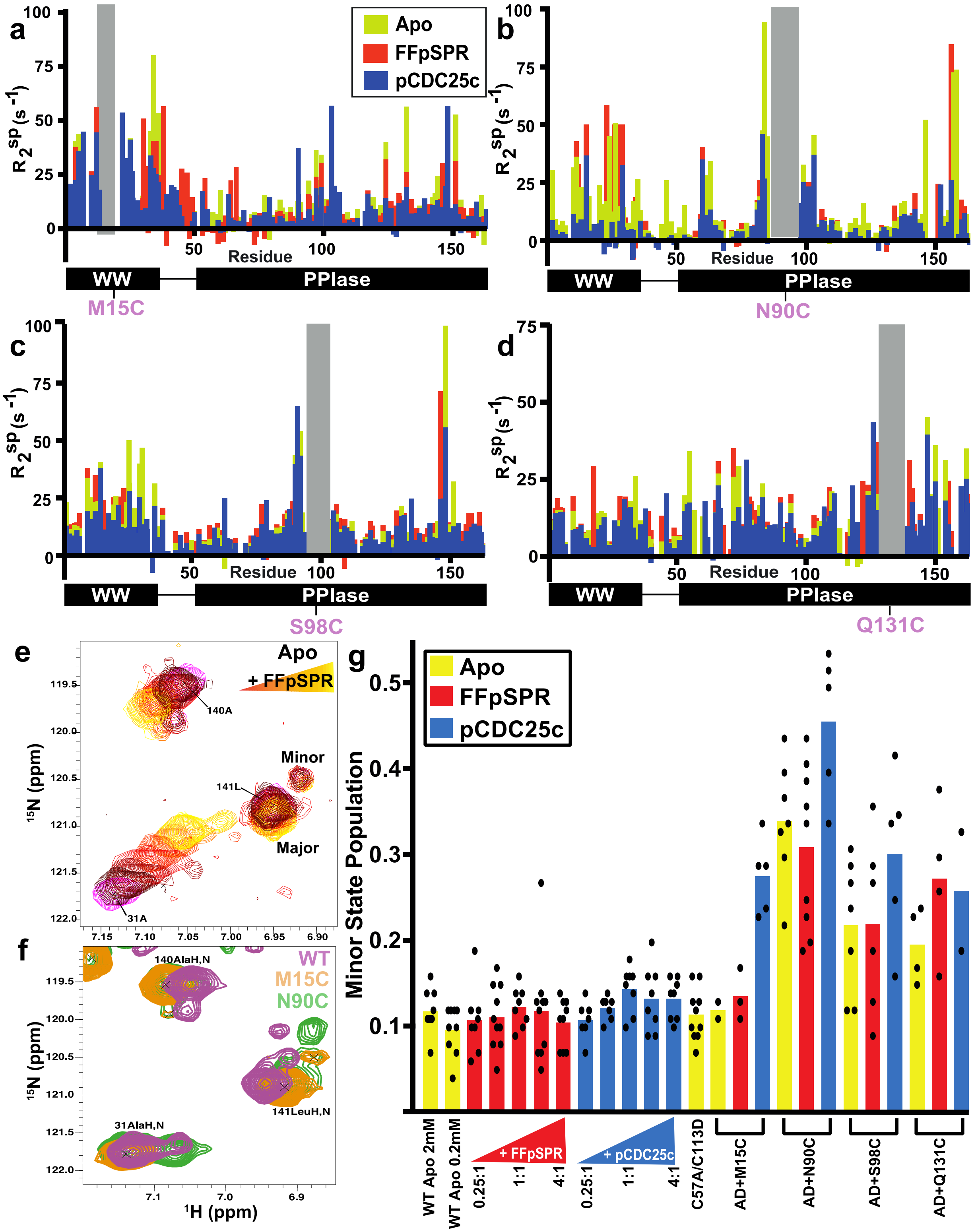

Supplementary Figure 3. **Impact of ligand binding on paramagnetic relaxation rates and slow-exchange interdomain interface peak intensities. a** - **d** PRE R_2^{sp} rates for spin labels 15-, 90-, 98-, and 131-MTSL, respectively. Grey bars indicate peak broadening beyond detection. **e** Major and minor interface peaks with various concentrations of FFpSPR. **f** Interface major and minor

peaks for WT, M15C, and N90C constructs. **g** Quantification of the most separate peak minor-state population (minor peak intensity/major + minor peak intensities) for various Pin1 conditions. The peak intensities were extracted from up to 10 residues within the interface, with the bar as the average intensity. The number of peaks plotted for each condition are as follows: 9, 9, 8, 10, 8, 10, 9, 7, 7, 8, 8, 8, 10, 2, 3, 5, 7, 9, 5, 7, 6, 5, 4, 4, 2.

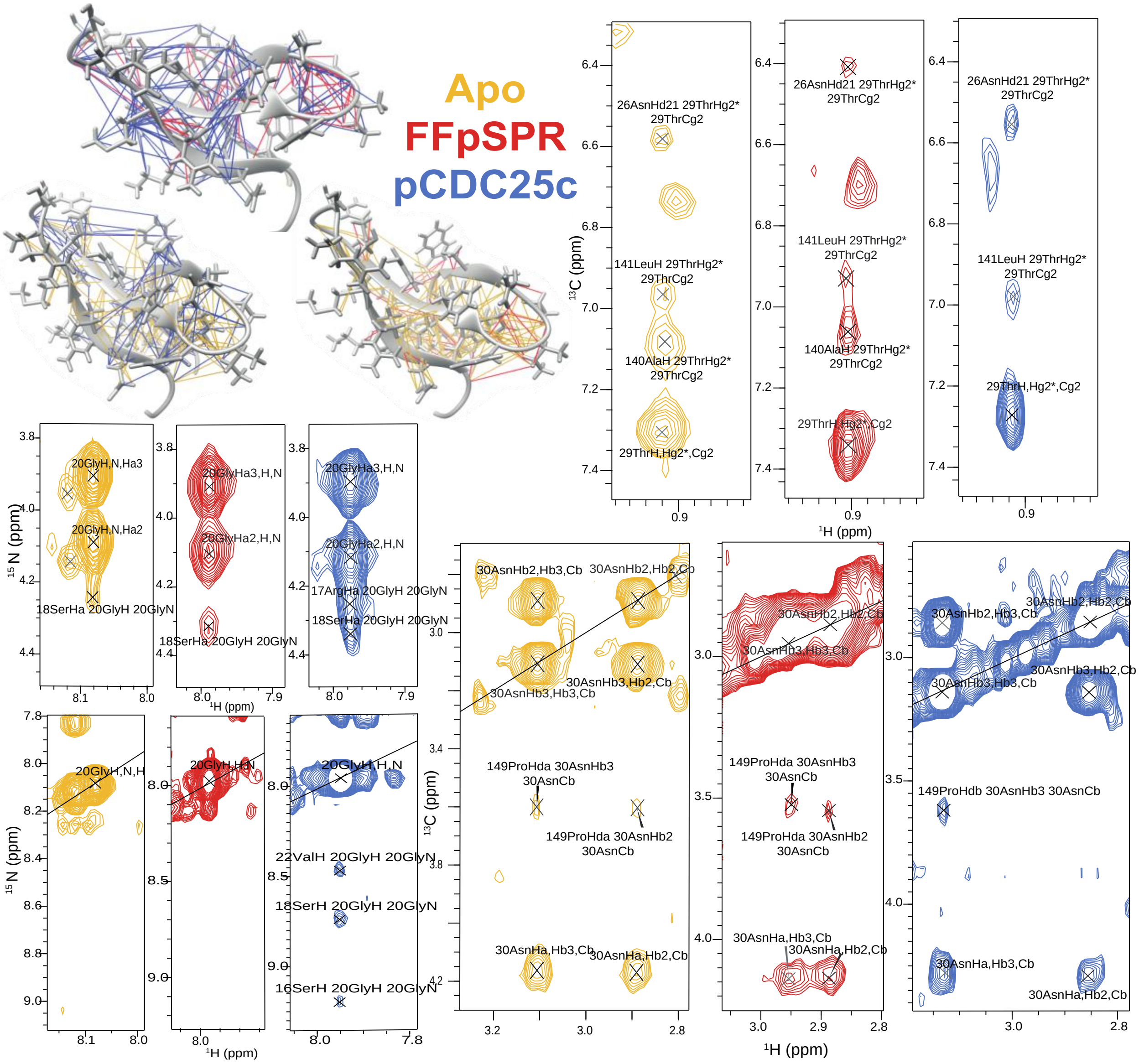

Supplementary Figure 4. **Impact of ligand binding on NOEs.** Differences in resolved, unique NOEs (exact- and generic-normalized) are plotted on the WW domain. The NOEs are compared between FFpSPR-bound and pCDC25c-bound (red/blue distance plot), between pCDC25c-bound and apo (blue/yellow), and between FFpSPR-bound and apo measurements (red/yellow), with the respective unique NOEs plotted. Example of changes in NOESY spectra among different protein complex measurements are shown.

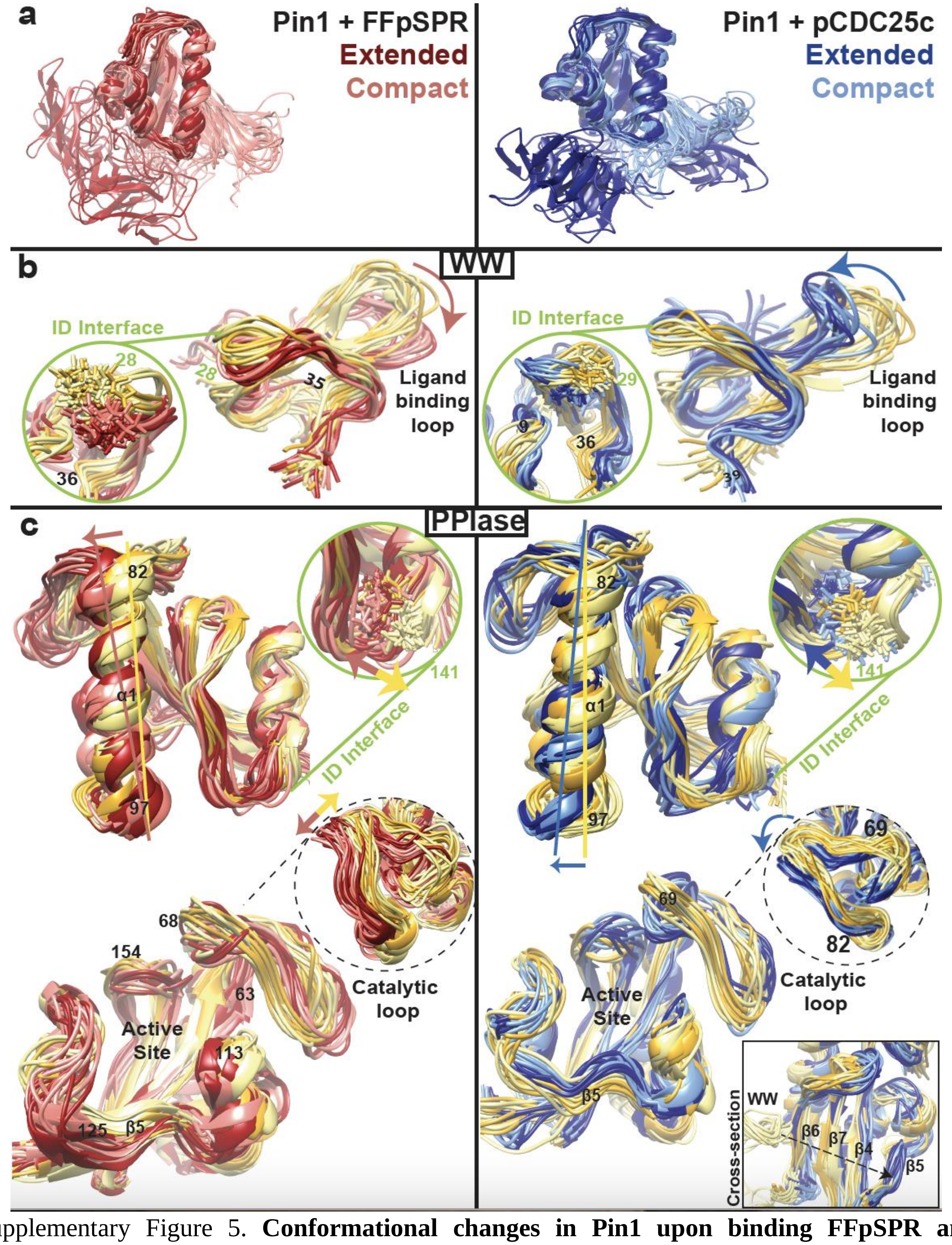

Supplementary Figure 5. **Conformational changes in Pin1 upon binding FFpSPR and pCDC25c. a** Two-state model of FFpSPR- and pCDC25c-bound Pin1 overlaid via the PPIase domain. By allowing two states to satisfy all intra- and inter-domain restraints, compact and extended states are observed. Ligand-dependent conformational changes seen within the two domains overlaid in the **b** WW and **c** PPIase domains. The two-state ensemble (goldenrod and khaki) of apo Pin1 is overlaid for comparison.

Supplementary Tables

Supplementary Table 1. **Tumbling times (τ_c) of Pin1 samples used in structure calculations.** Precise domain-specific τ_c values are necessary to calculate r_{eff} and determine eNOE distances. The values are given in units of nanoseconds.

	WW	Linker	PPIase
Apo	11.3	3.6	14.1
FFpSPR-bound	10.6	3.5	13.2
pCDC25c-bound	8.8	3.7	12.4

Supplementary Table 2. **Fit parameters for 4pDEER (pCDC25c-bound 15-98 mutant) and corrected 5pDEER (FFpSPR-bound 15-98 mutant).** Data is analyzed in terms of a model-free and a sum of two Gaussian distance distribution (see Supplementary Figure 2c and d). The values in brackets define the 95% confidence interval.

**mutant**	**ex_4pdeer**	**background**		**2Gauss**				
	λ	κ<br>[μs^{-1}]	d	$<r_1>$<br>[Å]	σ_1<br>[Å]	p_1	$<r_2>$<br>[Å]	σ_2<br>[Å]
15-98,<br>FFpSPR	0.14892<br>[0.14733<br>0.14976]	0.12499<br>[0.12399<br>0.12843]	0.90024<br>[0.90022<br>0.90043]	-	-	-	-	-
15-98,<br>FFpSPR	0.15215<br>[0.14373<br>0.15329]	0.11172<br>[0.1079<br>0.13053]	0.94085<br>[0.90001<br>0.95341]	24.014<br>[23.871<br>24.224]	7.4057<br>[6.2187<br>7.8165]	0.70363<br>[0.65077<br>0.72235]	46.148<br>[43.531<br>46.86]	18.609<br>[16.881<br>21.521]
15-98,<br>pCDC25c	0.28712<br>[0.28359<br>0.29421]	0.054178<br>[0.042695<br>0.058518]	0.91049<br>[0.90562<br>0.91326]	-	-	-	-	-
15-98,<br>pCDC25c	0.29941<br>[0.29389<br>0.29992]	0.02<br>[0.02<br>0.028129]	0.90044<br>[0.9<br>0.9037]	25.334<br>[25.235<br>25.444]	5.1669<br>[4.8875<br>5.5733]	0.66663<br>[0.64333<br>0.71712]	46.279<br>[44.549<br>48.398]	31.907<br>[25.679<br>33.936]

Supplementary Table 3. **Fit parameters for 5pDEER.** Data is analyzed in terms unparametrized and sum-of-two-Gaussians distance distributions for FFpSPR-bound 15-90 mutant and pCDC25c-bound 15-90 mutant (Supplementary Figure 2a and b), and model-free analysis of FFpSPR-bound 15-131 mutant and pCDC25c-bound 15-131 mutant (see Supplementary Figure 2e and f). The values in brackets define the 95% confidence interval.

**Mu-tant**	**ex_5pdeer**				**background**		**2Gauss**				
	Λ_0	λ_1	λ_2	$T_0^{(2)}$ [μs]	κ [μs^{-1}]	*d*	<r_1> [Å]	σ_1 [Å]	p_1	<r_2> [Å]	σ_2 [Å]
15-90, FFpSPR Fig. S5A	0.58557 [0.57588 0.59044]	0.32988 [0.32502 0.33803]	0.08723 [0.08524 0.09125]	2.9663 [2.9652 2.9675]	0.03304 [0.02000 0.03640]	0.91636 [0.9058 1.1086]	-	-	-	-	-
15-90, FFpSPR Fig. S5A	0.62044 [0.62556 0.63201]	0.35397 [0.35555 0.36261]	0.1055 [0.1048 0.10906]	2.9661 [2.9651 2.9674]	0.03249 [0.02971 0.03420]	0.91024 [0.90178 0.94247]	22.136 [22.081 22.184]	3.0004 [2.8658 3.1122]	0.73057 [0.71803 0.7422]	43.817 [43.078 44.536]	24.077 [22.453 26.018]
15-90, pCDC25c Fig. S5B	0.67088 [0.65954 0.67827]	0.27513 [0.26442 0.2843]	0.096558 [0.08812 0.10347]	2.9689 [2.9663 2.972]	0.03066 [0.02001 0.04702]	0.92343 [0.90046 1.0367]	-	-	-	-	-
15-90, pCDC25c Fig. S5B	0.65334 [0.64985 0.65797]	0.26641 [0.26455 0.27146]	0.10237 [0.09939 0.10616]	2.9691 [2.9665 2.972]	0.03605 [0.02331 0.03913]	0.91 [0.90198 1.101]	23.08 [22.888 23.243]	4.2691 [3.7818 4.9118]	0.50296 [0.47233 0.52711]	45.988 [45.293 46.743]	22.826 [20.9 25.205]
15-131, FFpSPR Fig. S6A	0.74612 [0.71596 0.74832]	0.20497 [0.20032 0.22057]	0.071835 [0.06819 0.08377]	3.2627 [3.2453 3.2795]	0.04617 [0.02662 0.04874]	0.91165 [0.90545 1.0283]	-	-	-	-	-
15-131, pCDC25c Fig. S6B	0.75495 [0.7391 0.75954]	0.18911 [0.18336 0.19617]	0.053168 [0.05086 0.05688]	3.7206 [3.7071 3.7512]	0.08261 [0.07361 0.09175]	0.96392 [0.92943 0.99448]	-	-	-	-	-

Supplementary Table 4. **Fit parameters for 4pDEER.** Data is analyzed in terms of unparametrized and single-Gaussian distance distributions for FFpSPR-bound 90-131 mutant and pCDC25c-bound 90-131 mutant (see Supplementary Figure 2g and h), and unparametrized analysis of FFpSPR-bound 15-131 mutant and pCDC25c-bound 15-131 mutant (see Supplementary Figure 2e and f). The values in brackets define the 95% confidence interval.

**mutant**	**ex_4pdeer**	**background**		**1Gauss**	
	λ	κ [μs^{-1}]	*d*	<*r*> [Å]	σ [Å]
90-131, FFpSPR	0.099928 [0.099047 0.10208]	0.075208 [0.067799 0.078509]	0.92178 [0.91946 0.92433]	-	-
90-131, FFpSPR	0.096411 [0.095235 0.098304]	0.097933 [0.082438 0.10189]	0.9 [0.9 1.0553]	26.616 [26.519 26.729]	4.1053 [3.8997 4.8717]
90-131, pCDC25c	0.20203 [0.19257 0.20866]	0.083555 [0.067897 0.11449]	0.99828 [0.93641 1.0415]	-	-
90-131, pCDC25c	0.18368 [0.18223 0.1856]	0.1389 [0.12867 0.14127]	0.9 [0.9 0.95467]	26.835 [26.774 26.9]	3.2503 [3.109 3.5979]
15-131, FFpSPR	0.26882 [0.26291 0.28314]	0.03943 [0.020438 0.047545]	0.91325 [0.90006 1.0435]	-	-
15-131, pCDC25c	0.23489 [0.23114 0.23778]	0.072225 [0.073321 0.081997]	1.0334 [0.98181 1.0267]	-	-

Supplementary Table 5. **Structural statistics and CYANA input data for apo, FFpSPR-bound, and pCDC25c-bound Pin1.**

NMR distance and dihedral constraints			
	**Apo Pin1***	**FFpSPR-bound Pin1**	**pCDC25c-bound Pin1**
**NOE distance constraints**			
Total eNOEs	2268	2456	2400
Bi-directional eNOEs	537	667	691
Uni-directional eNOEs	1731	1789	1709
gnNOEs	1937	894	1210
Interdomain NOEs	20	23	26
Intra-residue upl, $\|i-j\|=0$	569	571	615
Sequential upl, $\|i-j\|=1$	850	621	720
Medium-range upl, $1<\|i-j\|<5$	727	469	653
Long-range upl, $\|i-j\|\geq 5$	947	705	981
**Dihedral angle restraints**			
$^{3}J_{HNH\alpha}$	124	128	117
$^{3}J_{H\alpha H\beta}$	129	93	64
$^{3}J_{NC\gamma}$	12	11	13
Helix angle restraints ($\phi+\psi$)	66	66	66
**PRE/DEER interdomain restraints**			
Total ID PRE restraints	250	243	264
upl and lol	104	93	60
upl only	11	15	5
lol only	135	135	199
DEER restraints (r_{eff})	0	3	3
**Residual Dipolar Couplings**			
Total RDCs	407	0	0

	**Apo Pin1**		**FFpSPR-bound Pin1**		**pCDC25c-bound Pin1**	
	**1-state ensemble**	**2-state ensemble**	**1-state ensemble**	**2-state ensemble**	**1-state ensemble**	**2-state ensemble**
**Structure Statistics**						
CYANA target function (Å²)	316.3 ±7.6	148.3 ±3.9	297.8 ±1.8	206.8 ±5.4	268.6 ±2.4	146.0 ±2.7
NOE violations (>0.8 Å)	43	15	60	6	49	16
Scalar coupling violations (>2 Hz)	25	5	13	5	5	0
PRE violations (>6 Å)	30	7	21	15	14	12
**Deviations from mean**						
Backbone full WW (Å)	0.51 ±0.16	0.90 ±0.11	1.18 ±0.25	1.02 ±0.23	0.36 ±0.17	0.90 ±0.07
Backbone 2° elements WW (Å)	0.42 ±0.20	0.75 ±0.11	0.60 ±0.18	0.85 ±0.16	0.23 ±0.18	0.80 ±0.09
Backbone full PPIase (Å)	1.08 ±0.35	0.94 ±0.08	0.85 ±0.13	1.12 ±0.12	0.55 ±0.07	0.85 ±0.07
Backbone 2° elements PPIase (Å)	0.78 ±0.28	0.73 ±0.09	0.62 ±0.09	0.89 ±0.12	0.33 ±0.05	0.72 ±0.06

* the structure of apo Pin1 was previously published[5]

Figure References

1. Fábregas Ibáñez, L., Jeschke, G. & Stoll, S. Dipolar pathways in dipolar EPR spectroscopy. *Phys. Chem. Chem. Phys.* (2021). *Under review.*
2. Worswick, S. G., Spencer, J. A., Jeschke, G. & Kuprov, I. Deep neural network processing of DEER data. *Sci. Adv.* **4**, (2018).
3. Ranganathan, R., Lu, K. P., Hunter, T. & Noel, J. P. Structural and Functional Analysis of the Mitotic Rotamase Pin1 suggest substrate recognition is phosphorylation dependent. *Cell* **89**, 875–886 (1997).
4. Guo, J., Pang, X. & Zhou, H. X. Two pathways mediate interdomain allosteric regulation in Pin1. *Structure* **23**, 237–247 (2015).
5. Born, A. *et al.* Reconstruction of Coupled Intra- and Interdomain Protein Motion from Nuclear and Electron Magnetic Resonance. *J. Am. Chem. Soc.* **143**, 16055–16067 (2021).
